# Potential Activity of Fevicordin-A from *Phaleria macrocarpa* (Scheff) Boerl. Seeds as Estrogen Receptor Antagonist Based on Cytotoxicity and Molecular Modelling Studies

**DOI:** 10.3390/ijms15057225

**Published:** 2014-04-25

**Authors:** Muchtaridi Muchtaridi, Muhammad Yusuf, Ajeng Diantini, Sy Bing Choi, Belal O. Al-Najjar, Jerry V. Manurung, Anas Subarnas, Tri H. Achmad, Savitri R. Wardhani, Habibah A. Wahab

**Affiliations:** 1 Department of Pharmaceutical Analysis and Medicinal Chemistry, Faculty of Pharmacy, Universitas Padjadjaran, Jln. Raya Bandung Sumedang KM. 21, Jatinangor, West Java 45363, Indonesia; E-Mails: ajengdradjad@yahoo.com (A.D.); freemanusia@yahoo.com (J.V.M.); aasubarnas@yahoo.co.id (A.S.); 2 Pharmaceutical Design and Simulation Laboratory, School of Pharmaceutical Sciences, Universiti Sains Malaysia, 11800 Penang, Pulau Pinang, Malaysia; E-Mails: myusuf_setiabudi@yahoo.com (M.Y.); sybing@gmail.com (S.B.C.); najjar.belal@gmail.com (B.O.A.-N.); 3 Faculty of Medicine, Universitas Padjadjaran, Jln. Raya Bandung-Sumedang KM. 21, Jatinangor, West Java 45363, Indonesia; E-Mails: tachmad@fk.unpad.ac.id (T.H.A.); wsavitrirestu@yahoo.com (S.R.W.); 4 Malaysian Institute of Pharmaceuticals and Nutraceuticals, Ministry of Science, Technology and Innovation, Halaman Bukit Gambir, 11700 Penang, Pulau Pinang, Malaysia

**Keywords:** fevicordin A, estrogen receptor, pharmacophore, molecular dynamics, *Phaleria macrocarpa*

## Abstract

Fevicordin-A (FevA) isolated from *Phaleria macrocarpa* (Scheff) Boerl. seeds was evaluated for its potential anticancer activity by *in vitro* and *in silico* approaches. Cytotoxicity studies indicated that FevA was selective against cell lines of human breast adenocarcinoma (MCF-7) with an IC_50_ value of 6.4 μM. At 11.2 μM, FevA resulted in 76.8% cell death of T-47D human breast cancer cell lines. Critical pharmacophore features amongst human Estrogen Receptor-α (hERα) antagonists were conserved in FevA with regard to a hypothesis that they could make notable contributions to its pharmacological activity. The binding stability as well as the dynamic behavior of FevA towards the hERα receptor in agonist and antagonist binding sites were probed using molecular dynamics (MD) simulation approach. Analysis of MD simulation suggested that the tail of FevA was accountable for the repulsion of the *C*-terminal of Helix-11 (H11) in both agonist and antagonist receptor forms. The flexibility of loop-534 indicated the ability to disrupt the hydrogen bond zipper network between H3 and H11 in hERα. In addition, MM/GBSA calculation from the molecular dynamic simulations also revealed a stronger binding affinity of FevA in antagonistic action as compared to that of agonistic action. Collectively, both the experimental and computational results indicated that FevA has potential as a candidate for an anticancer agent, which is worth promoting for further preclinical evaluation.

## 1. Introduction

Breast cancer is the most common leading cause of cancer in women worldwide. According to the American Cancer Society, breast cancer represented 29% of cases (226,870) of all new cancer cases in the United States in 2012 [1]. In Indonesia, breast cancer is the second most common cancer in women after cervical cancer [2] and the incidence of breast cancer continues to increase. Whilst in Malaysia, it is the cancer with the highest prevalence (26.5%) in women compared to other cancer types such as cervix, uterine, colorectal, lung, and ovary [3].

There are a number of risk factors for breast cancer identified which include socioeconomic status [4–7] and genetic factors [8,9]. Family history, obesity or overweight, and oral contraception are also said to significantly contribute to the risk of developing breast cancer [2].

Experimental, clinical, and epidemiological evidences indicate that the sex hormones, estrogen and progesterone play an important role in cell growth and differentiation of breast cancer [10]. Furthermore, the presence of human estrogen and progesterone receptors in cancer cells is an essential key to guide therapy of breast cancer [11]. Estrogen receptors are found in both breast and colon cancers. Estrogen receptors (hERα and hERβ) have estradiol (E2) as their natural ligand, and data obtained from numerous *in vivo* observations indicated that E2 could promote breast cancer formation [12,13]. Quantum chemical calculations have previously showed the carcinogenicity of E2 [14]. The administration of 4-hydroxy tamoxifen (4OHT), which blocks hERα signaling thus reducing the cancer risk, also indirectly supported the role of E2 in breast cancer formation [15–17].

Researches in the last decade have accumulated a number of compounds derived from natural ingredients, referred to as phytoestrogens [18,19] such as daidzein, genistein [20], as well as glabridin [21] that can prevent cancer cell growth (antiproliferative). Although there have been debates about the carcinogenicity of phytoestrogens [22], such compounds were shown to bind to estrogen receptors stronger than E2 [21]. In this study, Fevicordin-A (FevA) isolated from mahkota dewa *Phaleria macrocarpa* (Scheff) Boerl. (or locally known as Mahkota Dewa) seeds was investigated for its potential as an anticancer agent. In Indonesia, this plant has been traditionally used as medicine for the treatment of human diseases including cancer, diabetes mellitus, and hypertension [23]. Anticancer effects or cytotoxicity of *P. macrocarpa* seeds have been previously reported against several human cancer cell lines (HT-29, MCF-7, HeLa and Chang cell lines) [24–26], however, the effect of any isolated compound of *P. macrocarpa* seeds that works against human breast cancer cell lines has yet to be reported. In this study, FevA isolated from *P. macrocarpa* seeds was investigated for its activity against human breast cancer cell lines. Furthermore, pharmacophore mapping, molecular dynamics simulation, and binding energy calculation using MM/GBSA were performed in order to study the antagonistic activity of this molecule on its possible receptor: hERα. This steroidal compound, also previously isolated from *Fevillea cordifolia* [25] was reported to show anti-inflammatory, cytotoxic, and antitumor activities [26].

## 2. Results and Discussion

### 2.1. Cytotoxicity of FevA on MCF-7 and T-47D Human Breast Cancer Cell Lines

**Figure 1.**
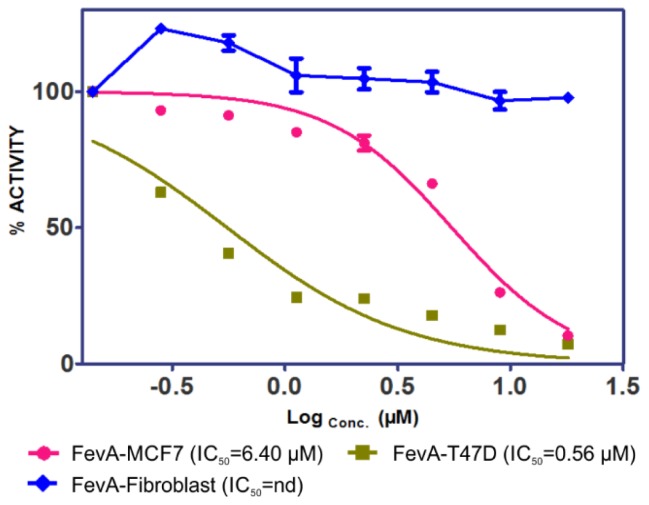
Cell proliferation inhibition profiles of FevA in MCF-7, T-47D, and fibrolast (as control) cell lines.

MCF-7 and T-47D human breast cancer cell lines known to contain hERα [27,28] were used in this study. The cells were exposed to different concentrations of FevA (11.23, 22.45, 44.90, 89.81, and 179.62 μM) after 24 h up to 48 h. The cytotoxicity of FevA was measured by IC_50_ calculated from the ratio of formazan absorbance, the product of MTT (3-(4,5-dimethylthiazolyl-2)-2,5- diphenyltetrazolium bromide) salt metabolism. Formazan was formed via the reduction of MTT in live mitochondria succinate reductase cells. Figure 1 shows the percentage of cell proliferation inhibition (CPI) due to FevA in MCF-7, T-47D, and human fibroblast cells (control).

The results showed a dose-dependent increase in the tumor cell death as compared to the control. It is very interesting to note that FevA was more selective for the tumor cells as demonstrated by the low cell death (<10% in all concentration tested) in the control. The percentage of cell death due to FevA in the breast cancer cells was more significant at the lowest concentration; for example at 11.23 μM, the percentages of death in MCF-7 and T-47D cell lines were 18.7% and 76.8%, respectively. The IC_50_ value of FevA in MCF-7 cells was 6.4 μM.

The results above imply that FevA has a potential to act against breast cancer. However, the mode of action of this compound in the breast cancer cells is not clearly defined. Due to the dominant presence of hERα in breast cancer cells and the E2-like ring structure of FevA, we assumed that the toxicity of FevA on the cells was probably due to the binding of the molecule onto the estrogen receptor, hERα. To support this hypothesis, we performed pharmacophore mapping and molecular dynamics simulation to study the antagonistic activity of this molecule on the receptor. Subsequently, MM/GBSA calculation from the MD simulation was carried out to study the binding affinity of FevA to the active site of the receptor.

### 2.2. Pharmacophore Mapping

**Figure 2.**
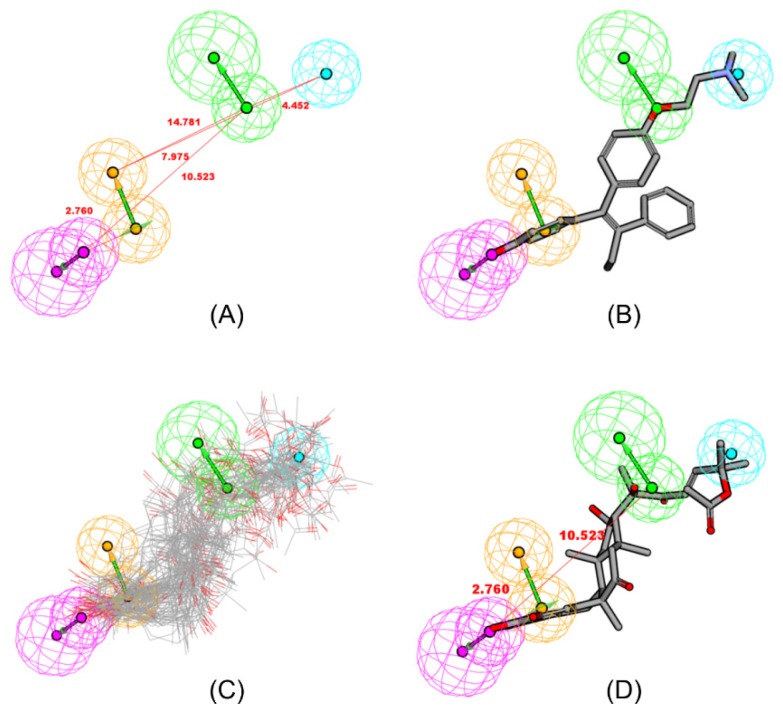
(**A**) HipHop1 model generated by HipHop module of Catalyst in DS 2.5 Package; Mapping of (**B**) 4OHT (4-hydroxy-tamoxifen) (**1**), the most-active compound (IC_50_ = 2 nM); and (**C**) Mapping of all conformations of FevA into HipHop1 model; (**D**) the best conformation of FevA, aligned into HipHop1 model. These features are color coded with hydrogen bond donor (HBD) in magenta, hydrogen bond acceptor (HBA) in green, hydrophobic feature (Hy) in blue and ring aromatic (RA) in orange.

The conformations of FevA were explored for all possible combinations of feature and interfeature distances. The similarity of the chemical feature distance between two ligands indicates similar biological properties [29]. From the training set, the best HipHop hypothesis (HipHop 1, Figure 2A), contained four features: one hydrogen-bond acceptor (HBA), one hydrogen-bond donor (HBD), one hydrophobic moiety (Hy), and one aromatic ring (RA). Figure 2B demonstrates the mapping of the best HipHop pharmacophore model against the most-active compound in the training set, *i.e.*, compound **1**, 4-hydroxytamoxifen, 4OHT (IC_50_: 2 nM). FevA satisfied the pharmacophoric features of a good antagonist for hERα, as all conformers of FevA mapped successfully into the pharmacophore model as shown in Figure 2C. (see also Supplementary Table S1).

The fit value represents how well the training compound is mapped to the generated hypothesis model. The higher fit value represents a better fit and a perfect mapping of features would result in a fit value equivalent to the sum of the weights of the features in the pharmacophore [30]. The best conformation of FevA (Figure 2D) showed that the distance between atom O-3 (HBD) and atom O-28 (HBA) is 10.52 Å, which was similar to the distance between O-3 (HBD) of E2 and O-17 (HBA) of E2 (10.90 Å). In addition, the distance between HBD and RA of FevA was 2.76 Å, which was similar to the distance between O-3 and the centre of ring A in the E2 structure. 4OHT (**1**) had the best fit on the model with the fit value (FV) of 3.998, whereas the FV of E2 was 1.990 as a result of missing two features as compared to FevA (FV = 3.481). It is a well known fact that the molecular features of agonists and antagonists are quite similar but differ only in a subtle change in atomic substitution. Therefore, this finding is not surprising as the model was derived from the antagonist training set while E2 is a well-known agonist for this receptor.

### 2.3. Molecular Dynamics of FevA on to ERα

**Figure 3.**
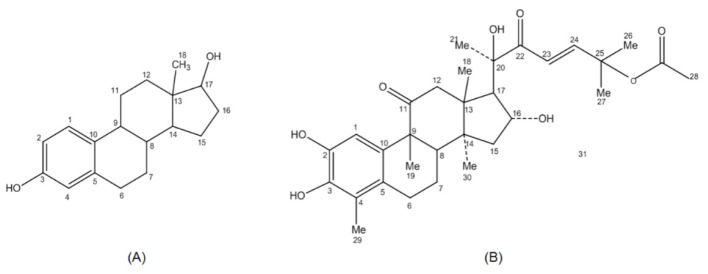
Chemical structures of E2 (**A**) and FevA (**B**).

FevA and E2 have a similar hydrophobic ring scaffold (Figure 3) despite their different structures. FevA has more hydrogen bond donors and acceptors than E2. E2 has only two hydroxyl groups at C2 and C17 while FevA has hydroxyl groups at C2, C3, C16 and C20. FevA also possesses carbonyl oxygens at C11 and C22, as well as an acetoxy group at the end of its tail. Therefore, FevA potentially has seven hydrogen bond acceptors and four hydrogen bond donors as compared to E2 which has two hydrogen bond acceptors and two hydrogen bond donors.

**Figure 4.**
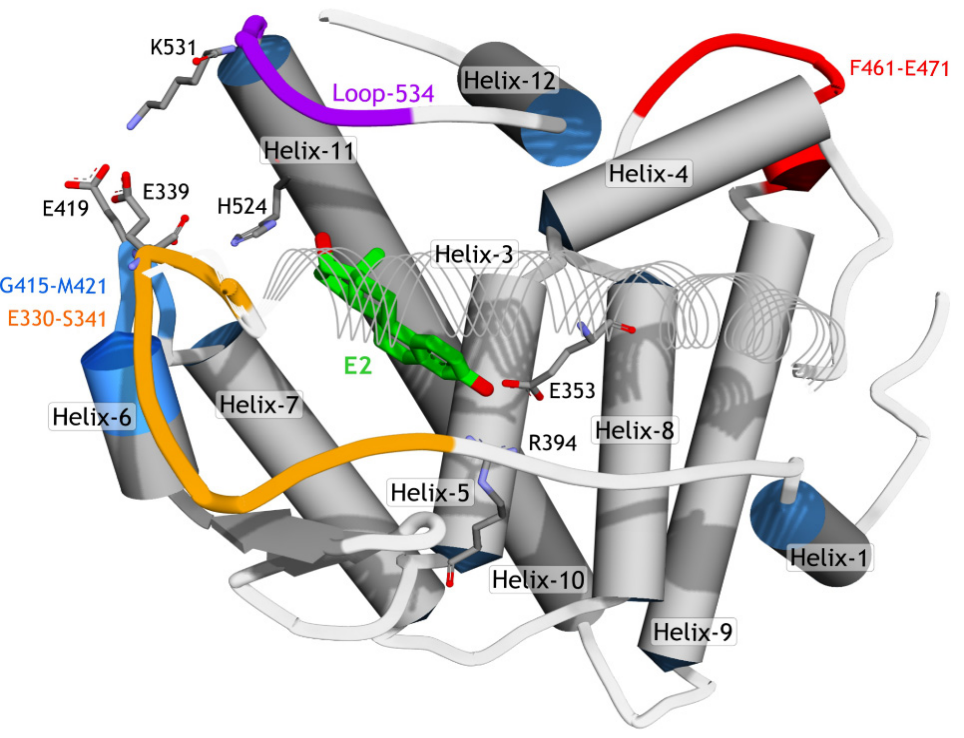
The schematic of hERα structure (PDB ID 1G50). To improve the visibility of the ligand (E2), Helix-3 is represented in line ribbon style.

A previous report of MD simulations (158 ns of time scale) on hERα [31] showed that the region between Helix-11 and Helix-12 (here noted as loop-534, Figure 4) was very flexible and thus was able to adopt different conformations such as the apo-, agonist-, and antagonist-forms. FevA has a similar skeleton with E2 (agonist), but its tail is similar to 4OHT (antagonist). For this reason, MD simulations for four systems, E2-1G50, FevA-1G50, 4OHT-3ERT, and FevA-3ERT, were performed. This work was intended to study the dynamic behavior of FevA inside the agonist’s and antagonist’s binding sites to ascertain a possible mechanism of action of FevA against hERα.

#### 2.3.1. Stability of the System

**Figure 5.**
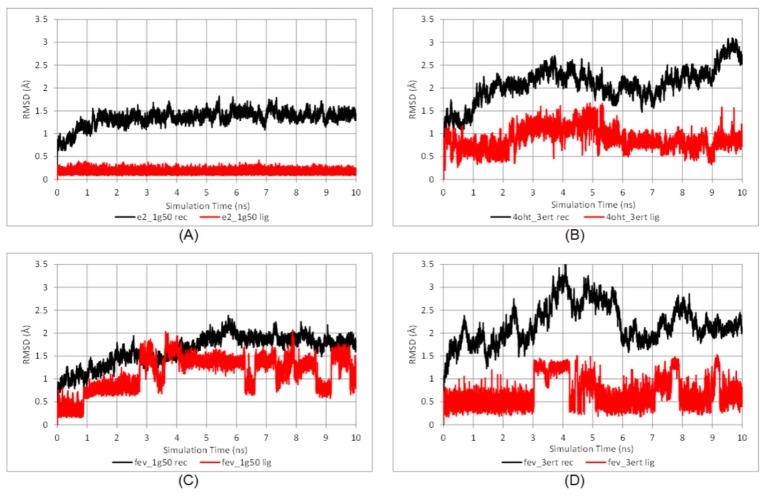
Time evolution RMSD of protein backbone (black line) and ligand (red line) throughout the 10 ns MD simulation time for (**A**) E2-1G50; (**B**) 4OHT-3ERT; (**C**) FevA-1G50; and (**D**) FevA-3ERT systems.

The thermodynamic properties (energies, temperature, volume, and pressure) of all systems shown in Supplementary Figure S1 reflected that the systems were well equilibrated. Time evolution RMSDs of the protein backbone and ligands during 10 ns of simulation are shown in Figure 5.

There was no significant deviation observed in the E2-1G50 system after the heating and equilibration steps. The RMSD became a plateau during the production period and remained around 1.5 Å for both the protein backbone and E2. As for the FevA-1G50 system, slight deviation of protein backbone occurred in the first 5 ns of simulation, but thereafter was stable with an RMSD of ~2 Å. In general, all the 3ERT systems showed higher deviations than 1G50, which is probably due to the higher flexibility of loop-534 in its open-conformation (3ERT) than in its closed ones (1G50).

**Figure 6.**
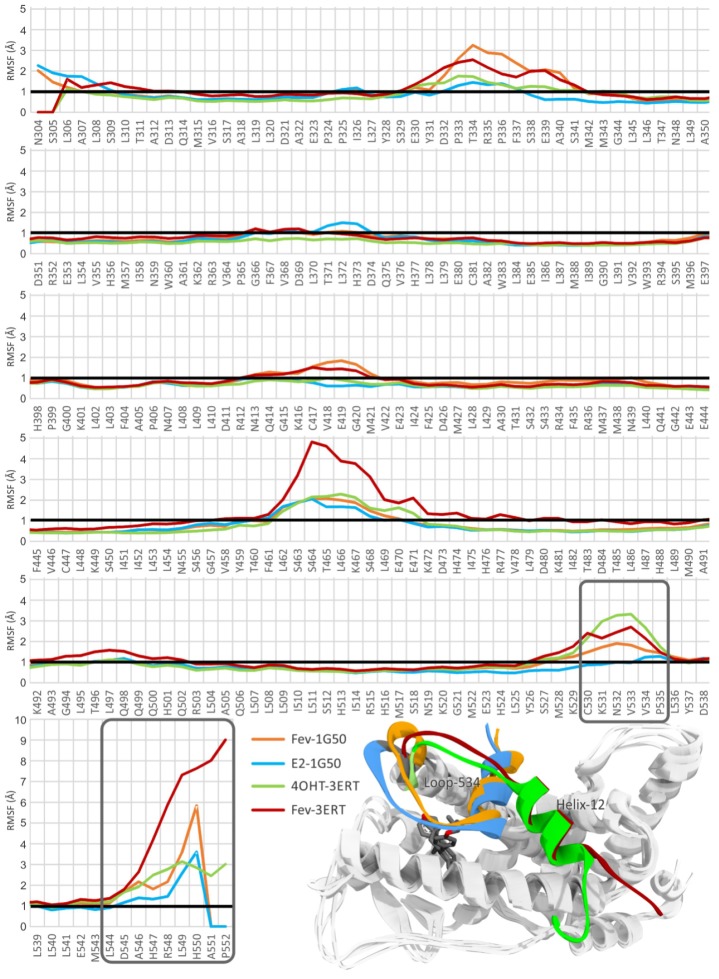
Average RMSF of amino acid residues of hERα in all the simulated systems. Inset is the superimposition of the average MD structures of E2-1G50, FevA-1G50, 4OHT-3ERT, and FevA-3ERT which are highlighted on the different conformations at Loop-534 and Helix-12 (blue, orange, green, and red colors, respectively).

The average RMSF values of amino acid residues 304–552 throughout the 10 ns simulations are shown in Figure 6. Significant fluctuation was observed mainly for the residues at the loop regions, *i.e.*, E330–S341, F461–E471, C530–P535, and those which are located at the *N-* and *C*-terminals. The results showed that the loops adopted high flexibility as compared to the other residues (particularly those in helices) in the protein. Interestingly, FevA and 4OHT appeared to increase the flexibility of the region of E330–S341, G415–M421 and C530–P535 (loop-534, Figure 4). The 1G50 crystal structure [32] revealed the hydrogen bond networks of E419–K531 and H524–E419. In the FevA-1G50 system, however, it can be seen that FevA disrupted this network and thus led to the increase of the flexibility of loop-534. This can be clearly seen in Figure 6 (inset) that shows loop-534 in the FevA-1G50 (orange colour) was repulsed by FevA, the behavior of which was also observed in the 4OHT-3ERT system (green colour). This behavior was not seen in the E2-1G50 system (blue colour). It is also of note that in the region of S463–E471 (located next to Helix-4 and Helix-12) of the FevA-3ERT system higher flexibility (>4 Å) was shown compared to that of the other systems (>2 Å).

**Figure 7.**
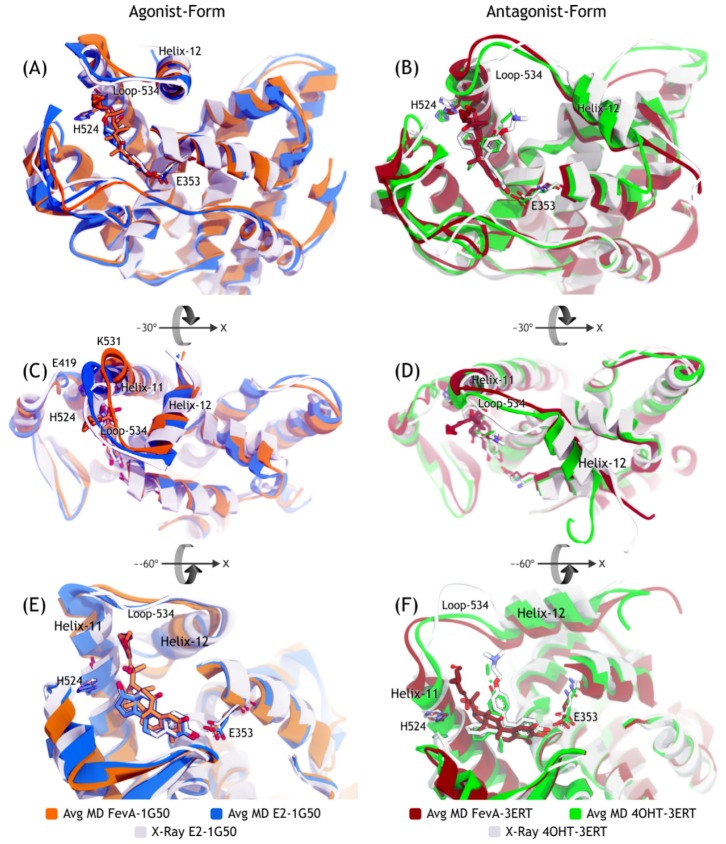
Superimposition of the average structures of all systems with the crystal structure. (**A**,**C**,**E**) are agonist-forms; (**B**,**D**,**F**) are antagonist-forms. (**C**,**D**) are the ~30° of *x*-axis rotation views from (**A**,**B**); while (**E**,**F**) are the ~−60° of *x*-axis rotation views from (**C**,**D**).

Superimpositions of the co-crystallized structures of 1G50 and 3ERT with the average MD structures are demonstrated in Figure 7. Figure 7A shows that there is no significant deviation in the average MD structure of E2-1G50 (blue colour) as compared to the starting crystal structure (grey colour). Meanwhile, Figure 7B shows a significant movement of loop-534 in 4OHT-3ERT (green colour) as compared to the starting crystal structure (grey colour). Interestingly, the presence of FevA in all systems affected the movement of loop-534 (orange colour in Figure 7C and red colour in Figure 7D) as compared to the starting crystal structures. Figure 7E shows that the basic skeleton of FevA had similar orientation to E2, where the C3 atom of FevA orientated near E353 and the tail (C17 atom) orientated close to H524. On the other hand, Figure 7F shows that the orientation of the FevA’s tail (red coloured stick) was different from that of 4OHT (green and white coloured sticks), which was located closer to the Helix-11 than the center of loop-534. Therefore, it can be proposed that the flexibility of E330–S341 and G415–M421 regions which was observed from RMSF values (Figure 6), might be a direct consequence of the movement of Helix-11, repulsed by FevA’s tail. This in turn led to the movement of loop-534, which further affected the conformation of Helix-12. Nevertheless, 10 ns may not be sufficiently long enough to observe this behavior, as it is evident that Helix-12 still remained at the same position for all the systems throughout the 10 ns simulations.

**Figure 8.**
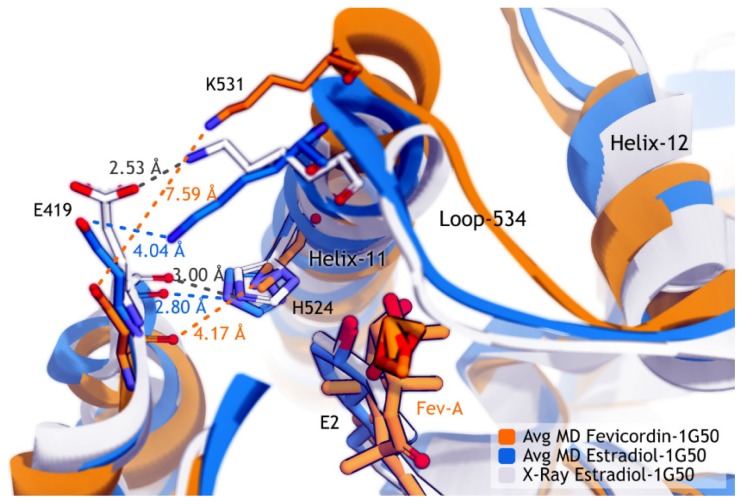
The superimposition of average MD structures of FevA-1G50 and E2-1G50 against the X-ray crystal structure of E2-1G50.

Figure 8 showed that K531 and E419 in 1G50 crystal structure formed a hydrogen bond at the distance of 2.53 Å. This distance was found to be longer in the average MD structures of E2-1G50 (4.04 Å) and FevA-1G50 (7.59 Å) systems. At the distance of 7.59 Å, minimum attractive force was retained between E419 and K531, which might explain the increased loop flexibility at the G415–M421 region. As a consequence, the distance between NE2 of H524 and O of E419’s main chain in the FevA-1G50 system also became further distanced apart (4.17 Å) compared to the other 1G50 systems (2.80 and 3.00 Å for E2-1G50 MD system and 1G50 crystal structure, respectively). An artifact which was found at the imidazole side chain of H524 in the average MD structure of FevA-1G50, (orange coloured stick at H524 in Figure 8) could have resulted from its flexible rotation during MD simulation. Furthermore, H524 was not as strongly stabilized by FevA as by E2, and this can be confirmed by the subsequent analysis of dihedral rotation of the H524’s side chain and hydrogen bond formation throughout 10 ns of MD simulations.

#### 2.3.2. Mode of Ligand Binding Interactions

**Figure 9.**
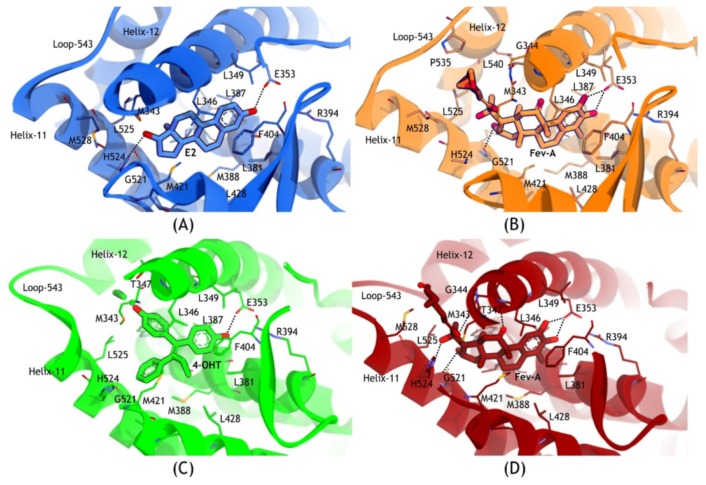
Interaction between (**A**) E2 with 1G50; (**B**) FevA with 1G50; (**C**) 4OHT with 3ERT; (**D**) 4OHT with 3ERT. E353 and H524 are noted as key residues in the interaction with E2 and FevA.

Figure 9 shows that most of the residues which interacted with E2, 4OHT and FevA were almost similar, strikingly made up of hydrophobic amino acids *i.e.*, M343, L346, T347, L349, A350, E353, L384, L387, M388, L391, R394, F404, M421, I424, L428, G521, H524, L525, and M528.

In addition, the tails of FevA and 4OHT had an additional interaction with W383. In the FevA-1G50 system, loop-534 was in close conformation which made more interactions with the tail of FevA through the residues V533, V534, P535, and L540. In all of the four systems, E353 had a conserved hydrogen bond with all the ligands. H524 also had a conserved hydrogen bond acceptor with E2 and FevA but not with 4OHT. Furthermore, in the FevA-3ERT system (Figure 9D), FevA was shown to form hydrogen bonds with T347, A350, and G521 as well as E353 and H524.

#### 2.3.3. Hydrogen Bonding Analysis

Hydrogen bond analysis from MD trajectories revealed that E353 was responsible for the formation of the hydrogen bond with all the studied ligands. The hydrogen bond’s percentage occupancy of E353 was always above 90% (Table 1).

**Figure 10.**
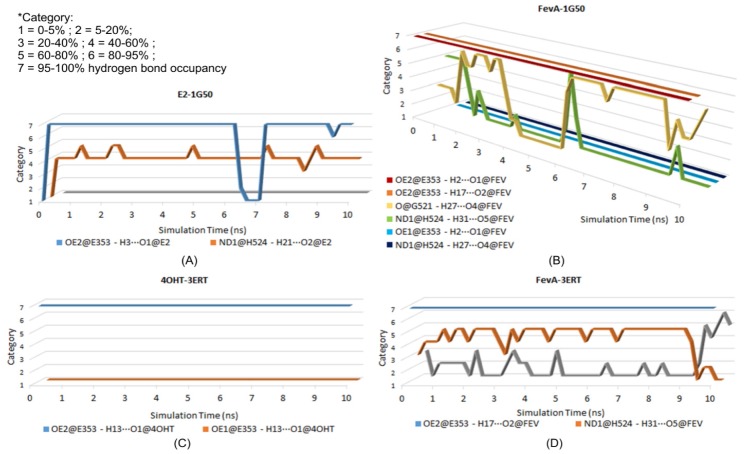
Time evolution plot of hydrogen bond occupancy for (**A**) E2-1G50; (**B**) FevA-1G50; (**C**) 4OHT-3ERT; and (**D**) FevA-3ERT systems throughout 10 ns of MD simulation. The shifting of hydrogen bonds between FevA with G521 and H524 are noted in both agonist and antagonist systems.

Table 1 shows that H524 also consistently formed a hydrogen bond with E2 and FevA. It is interesting to highlight that in the FevA-1G50 system (the agonist form), FevA tended to form a hydrogen bond with the oxygen carbonyl from the main chain of G521 (56.23%) instead of H524 (12.28%). This finding might explain the artifact of H524 in the average MD structure of the FevA-1G50 system (see above and Figure 8), that suggests H524 might not be stabilized by FevA, hence leads to the free rotation of its imidazole ring. On the other hand, surprisingly, FevA was found to have the tendency to form a hydrogen bond with H524 (59.2%) compared to G521 (13.81%) in the antagonist FevA-3ERT system. It is of note that the FevA’s atoms that were involved in hydrogen bonding with G521 and H524 are different, *i.e.*, H27···O4 and H31···O5, respectively. To get more understanding of this phenomenon, the time evolutions of the hydrogen bonding occupancy of all the systems were plotted in Figure 10.

The time evolution of the hydrogen bonding occupancy is defined into seven categories. Category 1, 2, 3, 4, 5, 6, and 7 are 0%–5%, 5%–20%, 20%–40%, 40%–60%, 60%–80%, 80%–95% and 95%–100% of hydrogen bond occupancy, respectively. Figure 10A showed that in the E2-1G50 system, the OE2 atom of E353 constantly formed a hydrogen bond with the O1 atom of E2 except for some length of times in between 6 and 7 ns (0%–5% occupancy) but increased to above 80% occupancy until the end of 10 ns of simulation. From the figure, there was also a small chance for the carboxylate group of E353 to rotate and allow its OE1 atom to form a hydrogen bond with E2 (0%–5% occupancy). On the other hand, Figure 10B showed that hydrogen bonds between FevA and G521 and H524 could not be formed simultaneously. At the first nanosecond, the hydrogen bond formed with H524 was more dominant than with G521. However, G521 consistently stabilized FevA in the binding site by the hydrogen bond until 4 ns. In between 5 and 6 ns, H524 regained its hydrogen bonding formation but later shifted again to G521. This implied that FevA required conformational adjustment to shift the hydrogen bond from G521 to H524 or *vice versa*. This phenomenon was also observed in FevA-3ERT (Figure 10D). At the final nanosecond of simulation, the hydrogen bonding occupancy of H524 had decreased and was replaced by the hydrogen bond with the main chain of G521. As for the 4OHT-3ERT system, the hydrogen bond between 4OHT and OE2 atom of E353 was observed to maintain 95%–100% of occupancy (Figure 10C).

#### 2.3.4. Dihedral Rotation of H524 Side Chain

An artifact structure of H524 shown in Figure 8 (FevA-1G50) and hydrogen bond analysis presented in Figure 10B suggest the instability of the H524 imidazole ring in the presence of FevA.

**Figure 11.**
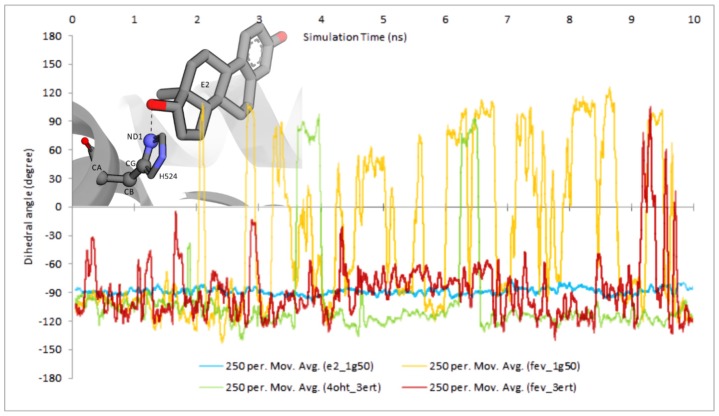
Time evolution of dihedral rotation of CA-CB-CG-ND1 angle in all the simulated systems throughout the 10 ns. The graph indicated that the side chain of H524 was stabilized in the presence of E2 and 4OHT. FevA appeared to destabilize the H524 especially in the agonist system (FevA-1G50).

Figure 11 showed the stability of H524’s CA-CB-CG-ND1 dihedral angle rotation in the E2-1G50 system throughout 10 ns of MD simulation, which is supported by the observation of consistent hydrogen bond formation between ND1 atom of H524 and O2 atom of E2. Moreover, the presence of FevA in 1G50 (agonist) indicated the free rotation of the H524 side chain which is in agreement with the observation of the artifact described above. It is suggested that FevA prefer to form a hydrogen bond mostly with G521, which lead to the free rotation of the H524’s side chain. Different phenomenon was observed however, in the FevA-3ERT system, where the rotation of the H524’s side chain only appeared at the end of 10 ns simulation. This is in agreement with the hydrogen bond analysis whereby the hydrogen bond shifted to G521 between 9 and 10 ns (Figure 10D). As for the 4OHT-3ERT system, the H524’s side chain remained stable despite the absence of hydrogen bond formation with 4OHT (Figures 10C and 11).

#### 2.3.5. MM/GBSA Calculation

Binding energies of FevA, E2, and 4OHT and their energy component breakdown are tabulated in Table 2. The intermolecular interaction (which is composed of van der Waals and electrostatic energies) of FevA with hERα outperformed the interaction with the other ligands, indicating that more interactions were formed between FevA and the receptor compared to either E2 or 4OHT. This is also corroborated by the fact that more hydrogen bonds (as explained in hydrogen bond analysis) and van der Waals interactions (as indicated from more surrounding residues) were formed by FevA with the receptor. Due to the lower number of hydrogen bond donors/acceptors in the main ring of 4OHT (one hydroxyl group) than in E2 (two hydroxyl group in C3 and C17), 4OHT has less electrostatic interaction as compared to E2. However, the structure of 4OHT is bigger than E2 thus leading to better van der Waals interaction. Moreover, most of the residues made up the binding site are hydrophobic amino acid residues.

Despite the entropy factor, FevA had better binding energy compared to E2 (−46.84 and −48.09 kcal/mol in agonist-form and antagonist form, respectively, compared to −34.80 kcal/mol), and slightly lower affinity than 4OHT with −50.16 kcal/mol. However, it is shown that FevA has a higher entropy factor compared to the others, which resulted in more positive binding energy (less affinity). The entropy of FevA might have resulted from the flexibility of its tail at the C17 position, which in turn contributed to more conformational changes of both FevA and its receptors during the simulation compared to the E2 and 4OHT systems, as reflected from the time evolution plot of RMSD values (Figure 5). Furthermore, the lower binding energy of FevA in the 3ERT receptor (−17.82 kcal/mol) than in the 1G50 (−12.49 kcal/mol) suggested that FevA prefers to bind in antagonist-form rather than agonist.

#### 2.3.6. Pairwise Decomposition of Binding Energy

The pairwise decomposition of binding energies (data shown in Supporting Information, Table S3) show that they mostly contributed from E353 and H524 (with the exception of E353 in the 4OHT-3ERT system). E353 was observed to have almost two-fold electrostatic interaction with FevA (~−12 kcal/mol) as compared to E2 and 4OHT (~−6 kcal/mol), which could have resulted from the interaction with two hydroxyl groups of FevA (at positions C2 and C3) as shown in Figure 9B,D. Figures 7E,F and 8 clearly showed that the tail of FevA pushed the end of Helix-11 away. Hence, FevA has more interactions with some residues from this region, *i.e.*, 525–528. Except for S527, the other three residues were shown to give an attractive force to the tail of FevA (Table S3).

### 2.4. Discussion

In Indonesia, the fruits and leaves of *P. macrocarpa* have been used empirically for cancer treatment. However, the seeds are very toxic, causing paraesthesia in the tongue, therefore they are only used externally such as for the treatment of skin diseases and as a biopesticide [23]. We discovered that the compound that may be the cause of toxicity in the seed was FevA. This compound exhibited toxicity against the brine shrimp (*Artemia salina*) and has cytoxicity on some human cancer cell lines such as P388, HeLa, CasKi, TE-2, and TE-8 cells [33]. The structural similarity between FevA and E2 has spurred renewed interest in studying the activity as an anti-cancer. Interestingly, this compound has an extension in C17 (Figure 3) compared to E2, which was mentioned as being a beneficial feature as hERα inhibitor [34].

Here, we showed that FevA isolated from *P. macrocarpha* seeds inhibited cell growth of breast cancer cell lines (MCF-7 and T-47D). Compared to its activity against normal cells (fibroblast) and human breast cancer cell lines, FevA might be more selective for the cancer cell lines thus it is predicted to have potential as an anti-breast cancer agent. It was observed that FevA activity against T-47D was very high even at the lowest concentration. However, the molecular level mechanism of FevA towards a specific receptor is still elusive and thus interesting to be elucidated. Estrogen receptors can be detected in about 70% of breast cancer cases. Breast cancers expressing estrogen or progesterone receptors respond well to hormonal therapy, implying that ligands which inhibit human ERα activity might be able to inhibit cancer cell growth.

In this study, *in silico* studies *i.e.*, pharmacophore modeling and molecular dynamics simulation were performed in our attempt to understand the chemical interaction of FevA with hERα. FevA has similar structural properties to E2 which is rigid but gains some flexibility from the rotation of groups attached to the C17 position. This compound has two hydroxyl groups in the steroidal ring, with an –OH group at the C3 position, which was shown to be important for binding to hERα.

Structural and functional analysis of E2 analogues suggested that structural conformation, substituent position, and physicochemical properties can influence the binding affinity [35,36]. These features can be seen in FevA as it also has a hydroxyl group at C2, methyl and carbonyl groups at C4 and C11, respectively (Figure 3) in addition to the isoheptenyl acetate at C17. FevA has HBD, HBA, Hy, and RA features similar to E2 and 4OHT derivatives. Moreover, the distance between O3–O20 (10.52 Å) of FevA was similar to the distance between O3–O17 of E2 which is comparable to the finding by Anstead *et al.* [37] (10.90 Å). The hydroxyl groups of FevA at C2, C3, C16, and C20 played a significant role in the formation of hydrogen bonds with the amino acid residues E353, G521, and H524 in hERα. This is especially the case with the hydroxyl groups at C2 and C3 which provided strong hydrogen binding interaction with E353, the key residue for the binding interaction of E2 or 4OHT with hERα. In addition, the hydroxyl group at C20 of FevA also contributed to the interaction with another important residue, H524.

The average MD structure of FevA-1G50 (agonist-form receptor) showed that FevA repulsed the *C*-terminal of Helix-11 which led to the disruption of the hydrogen bond network between E419, H524, and K531. Celik *et al.* [31] discovered that this hydrogen bond network is important to ensure that Helix-3 and Helix-11 are in close contact. Following this, the restrained Helix-11 would force Helix-12 to be positioned in the “mouse trap” as no other favorable (antagonist) conformation is available on the surface of the hERα ligand binding site. The investigation of the crystal structure of hERα in the antagonist-form complexed with 4OHT (PDB ID 3ERT [38]) revealed that this hydrogen bond network did not exist, which might be due to the antagonistic activity of 4OHT. Therefore, the broken hydrogen bond network in the agonist-form receptor due to the presence of FevA, might support the suggestion that FevA has antagonistic action. Moreover, H524 has also been postulated to have an important role in the formation of the hydrogen bond network with Helix-3 and Helix-11 [39]. However, based on the dihedral rotation and hydrogen bond analysis, H524 in the FevA-1G50 system was not well stabilized by FevA. The free rotation of the H524’s side chain in this MD system still occurred even though we had already assigned the protonation state of H524 in the ɛ-tautomer as suggested from the structural analysis of 1G50. This has also been well discussed as being the most stable tautomer in the previous MD simulation [31]. Thus, we suggest that the free rotation of H524’s side chain results from the presence of FevA, especially the shifting of hydrogen bond formation from G521–H27···O4 to H524–H31···O5 (and *vice versa*) during the 10 ns of MD simulation.

The repulsion of the *C*-terminal of Helix-11 where K531 is located by the tail of FevA resulted in the disruption of the hydrogen bond network between Helix-11 and Helix-3. Since Helix-11 was restrained, it led to the flexible movement of loop-534. This loop movement was closer to the conformation found in the antagonist systems (4OHT-3ERT MD system and the crystal structure of 3ERT). Although Helix-12 in the FevA-1G50 system consistently adopted the agonist position (“mouse trap”), we predict that the unrestrained Helix-11 and the movement of loop-534 will lead to conformational change of Helix-12 in extended MD simulation time. However, the current 10 ns time scale of MD simulation may not be sufficiently long to explore further this phenomenon. Finally, the MM/GBSA binding energy calculation, also supported the fact that FevA is more stable in the antagonist-form than in the agonist-form of hERα.

Katzenellenbogen [40] predicted that the volume of hERα’s ligand binding pocket is still larger (~450 Å^3^) than that occupied by E2 (~250 Å^3^). This indicates that there were some empty subpockets which were not filled by E2. One of these subpockets is at the 11β direction which represents the pocket for 4OHT’s bulky side chain that ends at the closed position of Helix-12. Katzenellenbogen [40] also suggested that these pockets could be enlarged even further to some degree, which might be the reason why this receptor is able to accommodate the tail of FevA.

The fluctuations of D545 in the FevA-1G50 and FevA-3ERT systems are similar to 4OHT-3ERT (Figure 6), but differ significantly from E2-1G50. Previously, D545 was found to be important to maintain the estrogen-like actions of the 4OHT-hERα complex [41]. Moreover, the pairwise decomposition of the binding energy shown in Table S3, indicates that FevA gained coulombic repulsion with D351 only in the FevA-1G50 (agonist) system. Kieser *et al*. [42] have suggested that the charge-charge repulsion with D351 is critical in distorting the position of Helix-12 and inducing ER degradation. Thus, it is suggested that FevA has a similar action with 4OHT as an antagonist.

The tail of FevA is attached at the position C-17 of the D-ring of the E2-like scaffold. The FevA’s tail is suggested to act with the side chains at the 11β position in a manner similar to that of 4OHT. It is interesting to note that the other E2 derivatives which have substitution at the 17α position act as agonists. The crystal structure of hERα complexed with 17R-(2E-trifluoromethylphenylvinyl) estradiol (PDB ID 2P15) [43] showed that the 17α side chain interacts towards Helix-7, in the opposite direction of Helix-11, Loop-534, and Helix-12. This might give a clue as to why FevA could act differently (as antagonist) compared to the other 17α substituted estradiol derivatives (as agonist) like those proposed in [44–46].

Furthermore, with regard to the tamoxifen (4OHT) resistance case [47], Levenson and Jordan [48] have noted that the majority (80%) of mRNA species in a tamoxifen-stimulated tumor have a D351Y mutation which is located at Helix-3 of hERα. The bulkier side chain of tyrosine compared to aspartic acid has resulted in the deflection of the antiestrogenic tail of raloxifene. It is worth noting from Figure 5F, the position of FevA’s tail is closer to Helix-11 than to Helix-3 (where D351 and E353 are located). Hence, it is suggested that FevA might be able to escape from the D351Y mutant.

## 3. Experimental Section

### 3.1. Materials

#### 3.1.1. Plant materials

*Phaleria macrocarpa* (Scheff.) Boerl was obtained from the Home Industrial Plants at Purworejo, Central Java, Indonesia and identified by the laboratory of Plant Taxonomy at Herbarium Bogoriense, Bogor, Indonesia. A voucher of the specimen was deposited at the Herbarium of the Bandung Institute of Technology, Bandung, Indonesia.

#### 3.1.2. Chemicals

Solvents for isolation: ethyl alcohol (EtOH), ethyl acetate (EtOAc), *n*-hexane, acetone, chloroform, and methanol (MeOH) (Merck, Darmstadt, Germany). For assay: fetal bovine serum (FBS) (Gibco), Leibovitz L-1, Roswell Park Memorial Institute medium (RPMI) 1640 (Invitrogen, Carlsbad, CA, USA), trypsin-Ethylenediaminetetraacetic acid (EDTA), l-glutamine, 4-(2-hydroxyethyl)-1- piperazineethanesulfonic acid (HEPES) buffer, sodium pyruvate (Invitrogen), Charcoal/dextran stripped FBS (C-SFBS) (Nissui, Tokyo, Japan), dimethyl sulfoxide (DMSO) (Sigma), Dulbecco’s modified eagle medium (DMEM) (Invirogen), Human ductal breast epithelial tumor cell line (T-47D), and human breast adenocarcinoma cell line (MCF-7) cancer cells (PAU-UGM).

#### 3.1.3. Equipment

Enzyme-linked immunosorbent assay (ELISA) (Bohreinger Mannheim GmbH, Mannheim, Germany), Spectrometers (Schimadzu, Kyoto, Japan), microplate 96-well plate, and microplate reader, centrifugation, and incubator (Memert, Schwabach, Germany).

#### 3.1.4. Software and Hardware

**Software:** Gaussian03 (Gaussian, Wallingford, CT, USA) [49], Accelrys Discovery Studio 2.5 (Accelrys Inc., San Diego, CA, USA) [50], Accelrys Discovery Studio Visualizer 3.5 (Accelrys Inc.) [51], AMBER 11 (Amber, San Francisco, CA, USA) [52], and AmberTools 1.5 (Amber) [52]. **Hardware:** 32-nodes Linux cluster, each powered by Intel Xeon 2.4 GHz processor and 2 GB of RAM (Intel, Santa Clara, CA, USA).

### 3.2. Methods

#### 3.2.1. Extraction and Isolation

The seeds of *Phaleria macrocarpa* (Scheff.) Boerl (1.8 kg) were extracted with EtOH. The EtOH extract (170.4 g) was partitioned between EtOAc and water to afford an active EtOAc extract (14 g) and was then chromatographed on Silica G-60 (CHCl_3_-MeOH in 10% steps) to obtain four active fractions. The fourth fraction (50 mg) was chromatographed on Silica G-60 with the same eluent. Further purification of active fractions on Silica G-60 was done with *n-*hexane-acetone (70:30). Recrystallization was carried out by MeOH thus producing 18 mg of FevA and confirmed by NMR data (Supplementary Table S2) [33].

#### 3.2.2. Assay

Cytotoxicity tests on MCF-7 and T-47D human breast cancer cells as well as on human fibroblast cells as a control were prepared according to MTT (3-(4,5-dimethylthiazolyl-2)-2,5-diphenyltetrazolium bromide) method [53]. The yellow tetrazolium MTT was reduced by metabolically active cells, in part by the action of dehydrogenase enzymes, to generate reducing equivalents such as NADH and NADPH. The resulting intracellular purple formazan was solubilised and quantified by spectrophotometric means.

#### 3.2.3. Cell Culture

Different lines of human breast cancer cells (T-47D and MCF-7) were originally obtained from the laboratory of (Interuniversity Centre (PAU), Gadjah Mada University, Yogyakarta, Indonesia). The cells were grown in DMEM supplemented with 2 mg/mL insulin, 1 mM sodium pyruvate, 1 mM non-essential amino acids, 4 mM glutamine, 10% FCS, and antibiotics (penicillin-streptomycin). One week before the experiment, the cells were transferred to phenol red-free medium supplemented with 5% C-SFCS.

#### 3.2.4. Isolation of Fibroblast Cells from Human Prepuce Skin

This step was performed in the LAF cabinet (Clyde Apac, Granville, Australia). Fibroblast primary cells were isolated from human male prepuce skin after circumcision. The fresh prepuce skin was soaked in povidoneiodium solution for 1 h and washed several times with phosphate buffer saline (PBS) for 30 min each. The dermis layer of prepuce skin was separated (2 mm × 2 mm size), cut into tiny pieces, and placed into culture flask. The cells were grown in high glucose Dulbecco’s Modified Eagle Medium (DMEM) which contained d-glucose, l-glutamine, sodium piruvate (Gibco) supplemented with 10% heat inactivated FBS (fetal bovine serum), penicillin (100 U/mL), streptomycine (100 U/mL) and fungi zone at 37 °C in 5% CO_2_ (Sanyo, Osaka Prefecture, Japan). The cells were differentiated by incubating them in their culture medium for 24 h and were collected the next day for further assay.

#### 3.2.5. Cytotoxycity Tests

Stock cultures were subcultured every 3–4 days using a trypsin 0.25%–EDTA 0.02% solution (Gibco BRL, New York, NY, USA). Cell viability was estimated by a modification of the MTT assay. Briefly, cells were plated in their growth medium at a density of 15,000 cells/well in 96 flat-bottomed well plates. Twenty-four hours after plating, FevA was added at concentrations ranging from 0.0115, 0.023, 0.046, 0.092 and 0.184 mM in DMSO. After 48 h incubation, the medium was replaced with MTT [54] (Sigma, St. Louis, MO, USA) dissolved at a final concentration of 1 mg/mL in serum-free, phenol-red-free medium for a further 4 h incubation. Then, the MTT-formazan was solubilised in isopropanol and the absorbance was measured at a wavelength of 450 nm.

Cell death (%)=(A450 (control)-A450 (sample))/A450 (control)×100%

where A450 is the absorbance at a wavelength of 450 nm, and the sample was the cancer cells with tested compounds.

#### 3.2.6. Pharmacophore Mapping

A conformational set was generated for each molecule using the polling algorithm and the best energy option, based on CHARMm force field [55] embedded in Discovery Studio 2.5 [50]. The molecules associated with their conformational models were mapped against the pharmacophore model using the “best fit” option. Qualitative HipHop models were built to identify the critical common essential chemical features and hence to provide information based on the four most-active compounds in the training set [56–58]. All the most-active compounds were considered with “principal” value of 2 and a “MaxOmitFeat” value of 0, the “Principal” values were set to 1 for the remaining 2 compounds.

#### 3.2.7. Molecular Dynamics Simulation

Four MD simulation systems were prepared in this study. The crystal structures of E2 (agonist) and 4OHT (antagonist) bound to hERα (Chain A of 1G50 and 3ERT, respectively) were used as the starting material. Some alternate conformations and incomplete residues in 3ERT were corrected using the “manual preparation” module in Accelrys Discovery Studio 2.5 [50]. All the hydrogen atoms were added explicitly using “tleap” module of the AmberTools 1.5 [52].

FevA 3D structure was prepared using Accelrys Discovery Studio 2.5 [50]. The parameterization for FevA was prepared following Duan *et al.* [59] which geometrically optimized at the HF/6–31** level followed by the electrostatic potential calculation at the B3LYP-IEFPCM/cc-pVTZ level with continuum solvent models and an effective dielectric constant of ɛ = 4 to mimic the interior of protein using Gaussian03 [49]. The electrostatic potential of FevA was fitted into partial atomic charge using the RESP method. Subsequently, the forcefield parameters were assigned using the Antechamber [60,61] module in Amber11 [52]. The optimized FevA structure was then superposed to the basic skeleton of co-crystallized E2/4OHT in PDB ID 1G50/3ERT, respectively, to form the complex starting structure.

The minimization of each complex was performed with the aid of Sander module in AMBER 11. Due to the bad steric clashes occurring particularly at the binding site in the starting Fev-hERα complexes, two stages of stepwise minimization were performed. Each system was minimized in the *in vacuo* condition prior to the solvation using both the steepest descent and conjugated gradient algorithm. AMBER FF03.r1 [59], all atom force field was used. All cysteines in the receptor were notated as CYS since there was no disulphide bridge formed. Each of the systems was immersed in TIP3P water box and neutralized with the addition of six sodium counter ions. PBC was applied with a non-bonded interaction cutoff at 9 Å. Then the complex was linearly heated to the physiological temperature at 310 K (0–100, 100–200, 200–310 K) in NVT ensemble using a Langevin thermostat with collision frequency of 1.0 ps^−1^ and harmonic restraint of 5 kcal·mol^−1^·A^−2^ on the backbone atoms. It was further equilibrated in NPT ensemble with harmonic restraint of 3 kcal·mol^−1^·A^−2^ and later 1 kcal·mol^−1^·A^−2^ for 2.5 ns. Finally, each of the systems was fully equilibrated without any restrain for 500 ps in the NPT ensemble and lastly the production runs were conducted under NPT conditions for 10 ns. The time steps used were 0.5 fs for the heating stage and 2.0 fs (with SHAKE applied) for the equilibration and production stages.

#### 3.2.8. Binding Energy Calculation

All binding energy calculations were done using a single trajectory approach using MMPBSA.py [62]. According to the MM/GBSA theory, binding free energy (Δ*G*_bind_) between a ligand (*L*) and a receptor (*R*) to form a complex RL is calculated as:

(1) $$\Delta G_{\text{bind}}=\Delta H-T\Delta S\approx \Delta E_{\text{MM}}+\Delta G_{\text{sol}}-T\Delta S$$

(2) $$\Delta E_{\text{MM}}=\Delta E_{\text{internal}}+\Delta E_{\text{electrostatic}}+\Delta E_{\text{vdw}}$$

(3) $$\Delta G_{\text{sol}}=\Delta G_{\text{GB}}+\Delta G_{\text{SA}}$$

where, *S* is the entropy, Δ*H* is the enthalpy and *T* is the temperature in Kelvin. Δ*E*_MM_ describes the molecular mechanical (MM) energy change in the gas phase while Δ*E*_int_ is the internal energy, Δ*E*_elec_ is the coulomb electrostatic term and Δ*E*_vdw_ is the van der Waals interaction term. Δ*G*_solv_ is the solvation free energy, Δ*G*_GB_ is the electrostatic solvation energy (polar contribution) calculated by GB model and Δ*G*_SA_ is the nonelectrostatic solvation component (nonpolar contribution).

The interval step of 10 ps for MM/GBSA calculation, and the salt concentration of 150 mM were applied. The residues around 5 Å from the ligand (from the average structure of each MD systems) were defined and their pairwise decomposition energies were calculated using MM/GBSA. As for the entropy calculation, quasiharmonic analysis was performed for each 0.2 ps.

## 4. Conclusions

The results showed that FevA has a potential activity against breast cancer cells, with an IC_50_ of 6.4 μM against MCF-7 cells. Moreover, FevA showed significant selectivity against T-47D human breast cancer cells over the control cells. Different pharmacophore models were generated using hERα antagonist data sets. Fit values obtained upon mapping the ligands against pharmacophore models showed that FevA and 4OHT satisfied the hERα antagonist pharmacophoric features, while E2 (agonist) showed significantly poor fitting. The effect of FevA upon binding within hERα was explained by the results of MD simulation. Hydrogen bond and RMSF analysis throughout 10 ns MD simulation showed that the binding of FevA and 4OHT (antagonist) onto hERα have similar effects towards loop-534, in which the movement resulted in the loss of agonist receptor conformation. Moreover, the MM/GBSA free energy of binding calculation revealed that FevA had a lower binding energy in the antagonist complex, (−17.82 kcal/mol) compared to the agonist complex, (−12.49 kcal/mol). Therefore, from the biological assay, pharmacophore mapping and molecular dynamics results, it can be proposed that FevA may act as an antagonist toward hERα thus warranting further investigation as a potential anticancer agent.

## Supplementary Information



## Figures and Tables

**Table 1.** Hydrogen bond analysis from 10 ns MD trajectories of all the studied systems.

System	H-Bond Acceptor (atom@res)	H-Bond Donor (H···atom@res)	Percentage Occupancy (%)	Average Distance (Å)	Average Angle (°)
E2-1G50	OE2@E353	H3···O1@E2	91.88	2.58	102.86
	ND1@H524	H21···O2@E2	54.41	2.87	103.06
	OE1@E353	H3···O1@E2	0.25	2.89	67.97

FevA-1G50	OE2@E353	H2···O1@FEV	99.88	2.60	106.21
	OE2@E353	H17···O2@FEV	99.01	2.65	107.55
	O@G521	H27···O4@FEV	56.23	2.77	100.46
	ND1@H524	H31···O5@FEV	12.28	2.85	103.73
	OE1@E353	H2···O1@FEV	0.28	2.93	65.59
	ND1@H524	H27···O4@FEV	0.03	2.89	82.24

4OHT-3ERT	OE2@E353	H13···O1@4OHT	99.34	2.64	98.74
	OE1@E353	H13···O1@4OHT	0.02	2.90	73.48

FevA-3ERT	OE2@E353	H17···O2@FEV	98.55	2.67	107.82
	ND1@H524	H31···O5@FEV	59.2	2.85	103.49
	O@G521	H27···O4@FEV	13.81	2.80	97.73

**Table 2.** The decomposition of calculated binding energies using MMGBSA.

Energy Component *	System			

	E2-1G50	FevA-1G50	4OHT-3ERT	FevA-3ERT
*Vdw*	−39.49	−62.73	−49.20	−56.63
EEL	−25.81	−43.72	−19.07	−42.80
ΔG_gas_ (*vdw* + EEL)	−65.30	−106.45	−68.26	−99.43
E_GB_	35.58	68.00	25.46	59.22
E_SURF_	−5.08	−8.39	−7.37	−7.88
ΔG_solv_ (E_GB_ + E_SURF_)	30.50	59.61	18.10	51.34
ΔG_MMGBSA_ (ΔG_gas_ + ΔG_solv_)	−34.80	−46.84	−50.16	−48.09
Entropy	−15.93	−34.35	−28.21	−30.27
ΔG_binding_ (ΔG_MMGBSA_ − Entropy)	−18.87	−12.49	−21.96	−17.82

* in kcal/mol.

## Abbreviations

MM/GBSA: Molecular Mechanics/Generalized Born Surface Area
RMSF: Root Mean Square Fluctuation
RMSD: Root Mean Square Distance
PDB: Protein Data Bank
hER: Human Estrogen Receptor
4OHT: 4-hydroxy tamoxifen
FevA: Fevicordin-A
CPI: Cell proliferation inhibition
MD: molecular dynamics
