# Supplementary material for: Potential Activity of Fevicordin-A from Phaleria macrocarpa (Scheff) Boerl. Seeds as Estrogen Receptor Antagonist Based on Cytotoxicity and Molecular Modelling Studies
Source: Int J Mol Sci. 2014 Apr 25;15(5):7225–49. doi: 10.3390/ijms15057225 (PMC4057669; doi:10.3390/ijms15057225)
Supplement: Supplementary file 1 [file ijms-15-07225-s001.pdf]

Supplementary Information

Table S1. Results of Model HipHop Pharmacophore.

No.	IC_{50} (nM)	Name of Compounds	Structure	Conf. Numbers	Fit Value	HBA	HBD	HY	RA	MaxOmit Feature	Principal
1	2.0	4-Hydroxytamoxifen	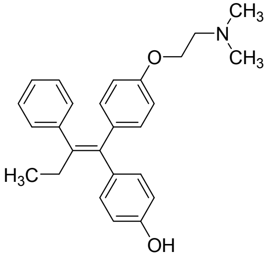	39	3.9997	1	1	1	1	0	2
21	0.5	Raloxifen derivative 5f	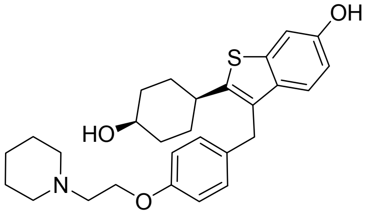	109	3.4799	1	1	1	1	0	1
2	1	Benzoxipen derivative 18	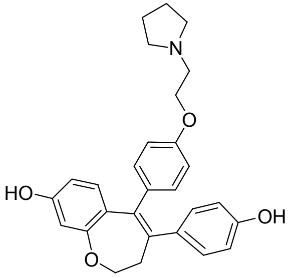	74	3.4925	1	1	1	1	0	1
3	1	Aril benzotiophene derivative	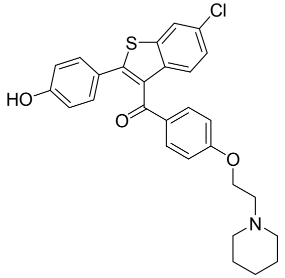	16	2.7051	1	1	1	1	1	1

HBA: Hydrogen Bond Acceptor; HBD: Hydrogen Bond Donor; Hy: Hydrophobic; RA: Ring Aromatics. MaxOmit: maximum omitted feature, Indicates how many features are allowed to miss for each molecule. 0 is used if the property is missing. Principal: Indicates whether the ligand is active (2), moderately active (1) or inactive (0). 2 is used if the property is missing.

Table S2. NMR data of FevA (400 MHz for ^{1}H NMR and 100 MHz for ^{13}C NMR in $CDCl_3$ solvent).

C Position	^{13}C NMR δ_C (ppm)	DEPT	^{1}H NMR, HMQC δ_H (ppm), [*mult., J* (Hz)]	HMBC ^{1}H - ^{13}C	Chemical Structure
1	109.1	CH	6.33 (1H, s)	C-2, C3, C9, C10	
2	141.1	Cq			
3	141.4	Cq			
4	123.0	Cq			
5	128.4	Cq			
6	23.6	CH_2	2.72 (1H, dd, 8.8; 18.4); 2.64 (1H, dd, 8.8; 18.4)		
7	19.2	CH_2	2.17 (1H, m); 1.87 (1H, m)	C-5, C10	
8	42.5	CH	2.02 (1H. m)		
9	50.9	Cq			
10	126.7	Cq			
11	216.4	Cq			
12	50.8	CH_2	2.88 (1H, d, 14.0); 2.67 (1H, d, 14.0)		
13	50.4	Cq			
14	48.7	Cq			
15	45.2	CH_2	1.95 (1H, m); 1.44 (1H, m)		
16	71.4	CH	4.31 (1H, dd, 7.2; 8.4)		
17	58.1	CH	2.43 (1H, d, 7.2)		
18	19.7	CH_3	0.98 (3H, s)	C12, C14, C13, C17	
19	28.9	CH_3	1.31 (3H, s)	C10, C9, C8, C11	
20	78.3	Cq			
21	23.8	CH_3	1.36 (3H, s)	C22	
22	202.6	Cq			
23	120.3	CH	6.96 (1H, d, 15.6)	C22, C25	
24	151.9	CH	7.00 (1H, d, 15.6)	C22, C25	
25	79.4	Cq	1.55 (1H, m)		
26	26.4	CH_3	1.46 (3H, s)	C24, C25	
27	25.7	CH_3	1.48 (3H, s)	C24, C25	
28	11.3	CH_3	2.02(1H, s)	C3, C4, C5	
30	19.6	CH_3	1.02 (1H, s)	C8, C14, C13, C15	
CH_3COO	170.5	Cq			
CH_3COO	21.9	CH_3	1.95 (1H, s)	CH_3COO	

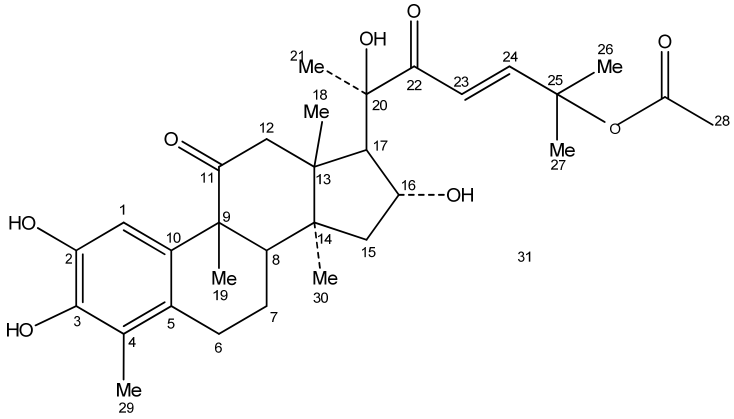

Table S3. Pairwise decomposition of binding energy between each binding site's residue with its respective ligand. (**A**) E2-1G50; (**B**) FevA-1G50; (**C**) FevA-3ERT; (**D**) 4OHT-3ERT. Unit of all values is in kcal/mol.

Table S3A

Residue	vdw	EEL	Polar solv	Nonpolar solv	Total
E353	1.11	−9.95	2.67	−0.30	−6.48
H524	−0.98	−2.13	0.38	−0.36	−3.09
L346	−1.66	−0.36	0.23	−0.48	−2.28
L387	−1.77	0.36	−0.28	−0.51	−2.21
L525	−1.35	−0.47	0.06	−0.43	−2.19
F404	−1.33	−0.25	0.21	−0.46	−1.84
L391	−1.24	−0.33	0.18	−0.43	−1.83
L384	−1.24	0.11	−0.18	−0.44	−1.76
M388	−1.54	0.22	0.13	−0.49	−1.69
M421	−0.95	−0.15	−0.06	−0.35	−1.52
M343	−0.68	−0.47	0.00	−0.32	−1.47
A350	−0.93	−0.10	0.13	−0.38	−1.29
G521	−0.90	0.28	−0.01	−0.35	−0.98
I424	−0.72	0.16	−0.07	−0.30	−0.93
L349	−0.75	0.11	0.12	−0.28	−0.81
T347	−0.65	−0.14	0.30	−0.26	−0.75
L428	−0.51	0.05	0.03	−0.25	−0.68
R394	−0.22	−0.54	0.34	−0.16	−0.58
W383	−0.23	0.08	−0.24	−0.08	−0.48
F425	−0.20	0.08	−0.14	−0.06	−0.32
M522	−0.24	0.08	0.01	−0.10	−0.26
M528	−0.13	−0.01	0.02	−0.08	−0.20
E385	−0.06	0.55	−0.68	0.00	−0.19
I386	−0.07	0.11	−0.14	0.00	−0.10
E523	−0.07	−0.03	0.01	0.00	−0.09
D351	−0.06	−0.09	0.07	0.00	−0.08
D426	−0.01	−0.12	0.05	0.00	−0.08
G344	−0.03	0.00	−0.03	0.00	−0.05
K520	−0.13	0.20	−0.07	−0.05	−0.04
V392	−0.06	−0.12	0.16	0.00	−0.02
W393	−0.02	−0.05	0.05	0.00	−0.01
V422	−0.03	0.03	−0.01	0.00	0.00
E423	−0.02	−0.25	0.29	0.00	0.01
L345	−0.08	−0.06	0.15	0.00	0.02
M427	−0.03	0.04	0.02	0.00	0.04
S527	−0.03	0.04	0.04	0.00	0.05
N348	−0.07	−0.01	0.13	0.00	0.06
Y526	−0.05	0.07	0.04	0.00	0.07
R352	−0.04	0.60	−0.48	0.00	0.08

Table S3B

Residue	vdw	EEL	Polar solv	Nonpolar solv	Total
E353	1.98	−16.94	3.37	−0.34	−11.93
M343	−2.51	0.04	−1.19	−0.74	−4.40
T347	−2.51	−0.88	0.83	−0.69	−3.26
H524	−1.81	−0.80	0.04	−0.49	−3.06
F404	−1.55	−0.50	0.08	−0.50	−2.47
G521	−0.61	−2.26	0.79	−0.37	−2.44
L346	−2.07	0.20	0.15	−0.53	−2.25
M421	−1.04	−0.29	−0.46	−0.36	−2.14
L525	−1.73	0.08	0.10	−0.54	−2.09
L384	−1.25	0.06	−0.25	−0.47	−1.91
M528	−1.18	−0.19	0.23	−0.43	−1.57
M388	−1.53	0.25	0.20	−0.49	−1.56
L391	−1.33	−0.17	0.38	−0.43	−1.55
A350	−1.39	−0.95	1.38	−0.49	−1.46
P535	−1.25	0.06	0.56	−0.46	−1.09
L387	−1.16	0.23	0.24	−0.39	−1.08
L349	−0.80	−0.88	0.95	−0.25	−0.99
G344	−0.55	0.28	−0.44	−0.23	−0.94
W383	−0.29	0.03	−0.57	−0.09	−0.92
Y537	−0.37	−0.01	−0.32	−0.13	−0.82
I424	−0.96	0.10	0.47	−0.35	−0.74
M522	−0.43	−0.14	0.01	−0.16	−0.72
L540	−0.75	−0.03	0.38	−0.29	−0.70
D351	−0.12	0.36	−0.79	0.00	−0.55
F425	−0.20	−0.01	−0.22	−0.04	−0.47
L428	−0.57	0.04	0.35	−0.27	−0.44
E385	−0.07	−0.18	−0.18	0.00	−0.43
D538	−0.03	−0.18	−0.12	−0.01	−0.34
D426	−0.02	−0.05	−0.27	0.00	−0.34
V534	−0.53	−0.01	0.55	−0.21	−0.20
V533	−0.54	−0.02	0.61	−0.24	−0.20
L536	−0.15	0.04	0.04	−0.07	−0.15
E523	−0.11	−0.50	0.52	−0.01	−0.11
Y526	−0.08	0.04	−0.01	0.00	−0.05
W393	−0.02	0.00	−0.03	0.00	−0.04
G390	−0.07	−0.08	0.13	−0.02	−0.04
I389	−0.07	0.03	0.05	−0.01	0.00
E423	−0.04	−0.31	0.37	0.00	0.02
V422	−0.03	−0.02	0.17	0.00	0.11
I386	−0.07	0.03	0.22	0.00	0.18
L345	−0.16	0.03	0.32	−0.01	0.19
S527	−0.06	0.07	0.19	0.00	0.20
L539	−0.03	−0.03	0.27	0.00	0.21
M427	−0.03	0.02	0.24	0.00	0.22
N348	−0.15	−0.17	0.73	−0.02	0.39
V392	−0.06	−0.03	0.56	−0.01	0.47
R352	−0.06	−0.38	1.04	0.00	0.61
R394	−0.26	0.67	0.49	−0.16	0.75

Table S3C

Residue	vdw	EEL	Polar solv	Nonpolar solv	Total
E353	0.72	−8.73	2.74	−0.29	−5.56
L346	−2.65	−0.52	0.14	−0.69	−3.72
L525	−1.80	0.01	−0.12	−0.67	−2.59
L387	−1.92	0.36	−0.45	−0.55	−2.55
L391	−1.15	−0.27	−0.09	−0.46	−1.97
T347	−1.59	−0.28	0.49	−0.46	−1.83
M388	−1.34	−0.04	0.12	−0.51	−1.75
A350	−0.98	−0.01	−0.15	−0.51	−1.64
L384	−1.18	0.15	−0.14	−0.44	−1.62
M421	−0.99	−0.07	0.11	−0.40	−1.34
L349	−0.92	0.03	−0.12	−0.32	−1.33
M528	−0.84	−0.15	0.00	−0.33	−1.32
M343	−1.13	0.10	0.14	−0.41	−1.29
I424	−0.78	0.09	−0.21	−0.35	−1.24
W383	−0.89	0.07	0.06	−0.34	−1.10
H524	−0.75	0.02	−0.08	−0.28	−1.09
G521	−0.50	−0.37	0.09	−0.29	−1.07
K529	−0.54	0.04	−0.32	−0.22	−1.03
F404	−0.77	−0.17	0.33	−0.35	−0.96
L428	−0.44	−0.02	−0.07	−0.26	−0.79
R394	−0.40	0.75	−0.64	−0.21	−0.49
K520	−0.10	−0.26	0.05	−0.05	−0.37
G390	−0.08	−0.19	−0.03	−0.03	−0.33
L345	−0.11	−0.13	−0.07	0.00	−0.31
L402	−0.12	−0.04	−0.05	−0.07	−0.28
M522	−0.23	−0.03	0.12	−0.12	−0.25
I386	−0.07	0.10	−0.27	0.00	−0.25
V392	−0.05	−0.08	−0.10	0.00	−0.24
N348	−0.11	−0.07	−0.01	0.00	−0.19
R352	−0.05	0.68	−0.76	0.00	−0.14
D351	−0.19	−0.78	0.91	−0.06	−0.12
L403	−0.04	0.04	−0.10	0.00	−0.10
E523	−0.06	0.10	−0.12	0.00	−0.08
I389	−0.06	−0.01	−0.01	0.00	−0.07
G344	−0.09	−0.02	0.04	−0.01	−0.07
E423	−0.03	0.14	−0.17	0.00	−0.06
S527	−0.03	−0.02	0.01	0.00	−0.04
V422	−0.03	0.01	−0.01	0.00	−0.03
E385	−0.06	0.47	−0.43	0.00	−0.02
W393	−0.01	−0.01	0.04	0.00	0.01
Y526	−0.06	0.01	0.09	0.00	0.04

Table S3D

Residue	vdw	EEL	Polar solv	Nonpolar solv	Total
E353	1.78	−16.99	3.39	−0.33	−12.15
H524	−2.21	−2.87	0.34	−0.59	−5.32
T347	−2.95	−0.39	−0.37	−0.79	−4.50
M343	−2.13	0.36	−1.46	−0.64	−3.87
M528	−1.89	0.04	−0.21	−0.61	−2.67
F404	−1.56	−0.54	0.13	−0.50	−2.47
L346	−1.83	0.09	−0.01	−0.50	−2.25
L525	−1.67	−0.27	0.29	−0.45	−2.11
L384	−1.36	−0.02	−0.20	−0.48	−2.06
M421	−0.82	−0.17	−0.36	−0.31	−1.65
M388	−1.55	0.20	0.24	−0.49	−1.59
G521	−0.96	−0.69	0.46	−0.36	−1.55
L391	−1.30	−0.18	0.41	−0.43	−1.50
A350	−1.48	−0.69	1.24	−0.51	−1.45
L387	−1.30	0.20	0.30	−0.42	−1.23
G344	−0.51	0.07	−0.46	−0.24	−1.13
W383	−0.38	0.00	−0.50	−0.14	−1.02
L349	−0.80	−0.73	0.87	−0.25	−0.92
D351	−0.14	−0.11	−0.37	0.00	−0.63
S518	−0.13	0.01	−0.49	−0.01	−0.62
I424	−0.74	0.10	0.36	−0.30	−0.58
M522	−0.31	0.01	0.02	−0.11	−0.39
E385	−0.07	0.04	−0.32	0.00	−0.35
L428	−0.44	0.03	0.32	−0.22	−0.31
G390	−0.07	−0.08	0.13	−0.02	−0.04
W393	−0.02	0.00	−0.02	0.00	−0.03
I389	−0.07	0.02	0.05	−0.01	−0.01
Y526	−0.09	0.03	0.05	0.00	−0.01
E523	−0.10	−0.55	0.65	0.00	−0.01
S527	−0.09	0.01	0.08	−0.01	0.00
E423	−0.04	−0.39	0.50	0.00	0.08
L345	−0.15	0.04	0.21	−0.01	0.10
V422	−0.03	0.01	0.12	0.00	0.10
N519	−0.04	−0.03	0.23	0.00	0.16
I386	−0.07	0.02	0.26	0.00	0.21
N348	−0.18	−0.13	0.55	−0.02	0.21
V392	−0.06	−0.03	0.54	−0.01	0.44
R352	−0.06	−0.09	0.77	0.00	0.63
K520	−0.16	0.40	0.43	−0.04	0.63
R394	−0.40	1.52	0.14	−0.22	1.04

Figure S1. The good stabilities of (**A**) E2-1G50; (**B**) FEV-1G50; (**C**) 4OHT-3ERT; and (**D**) FEV-3ERT systems are indicated by potential energy (EPTOT), kinetic energy (EKTOT), total energy (ETOT), temperature (TEMP), volume, and pressure over simulation time. EPTOT, EKTOT, and ETOT units are kcal/mol. Units for TEMP, VOLUME, and PRESSURE are kelvin, Å^3, and bar, respectively.

(A)

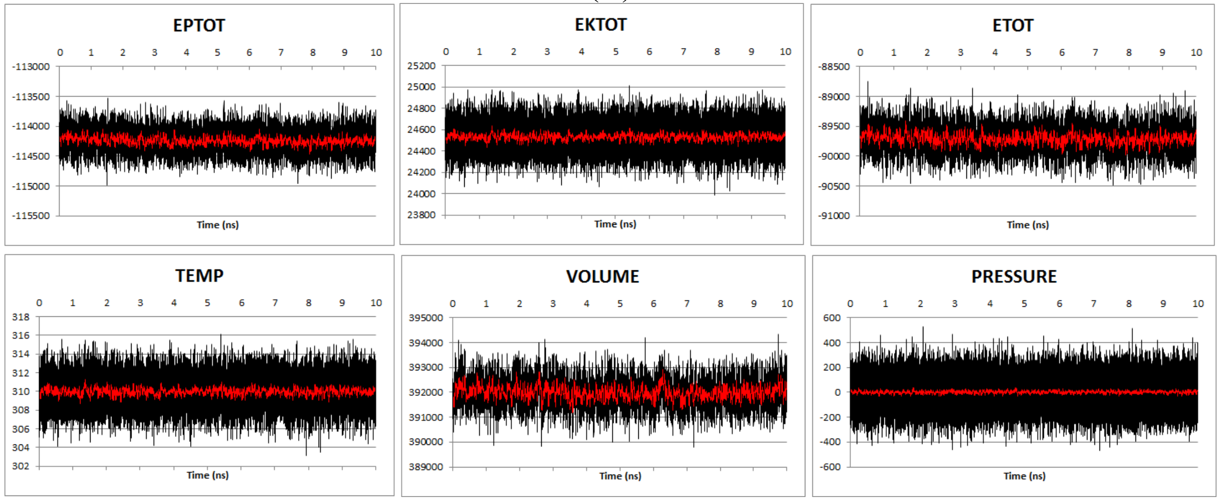

(B)

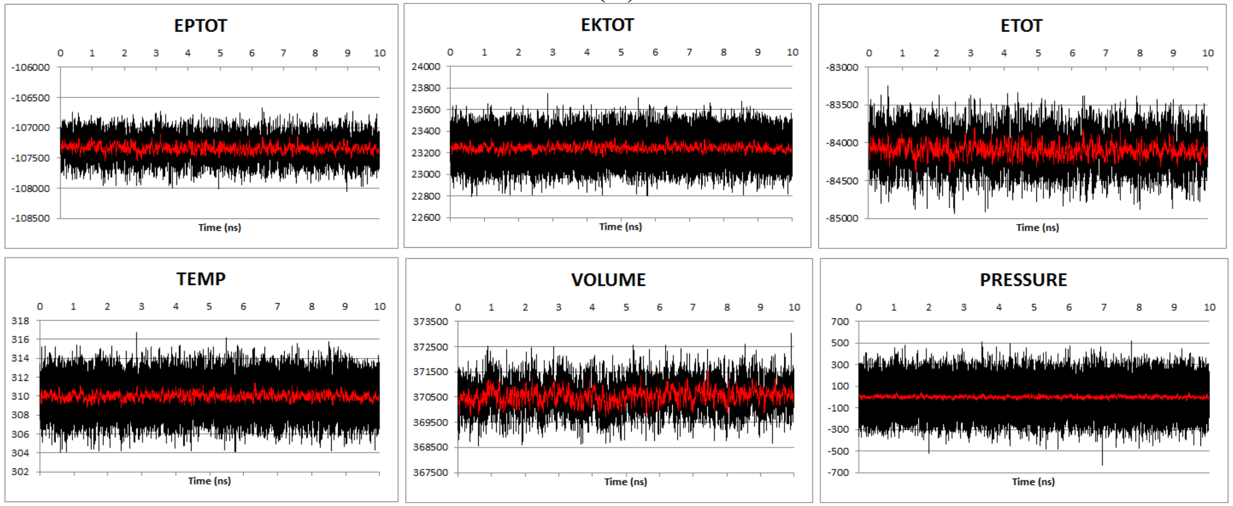

Figure S1. *Cont.*

(C)

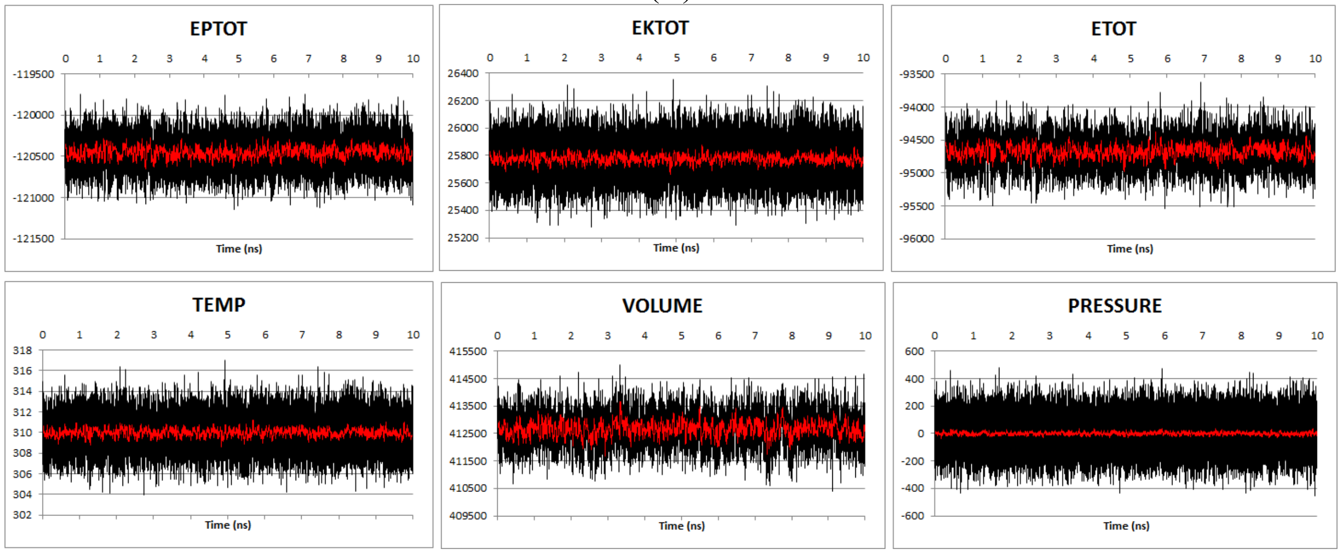

(D)

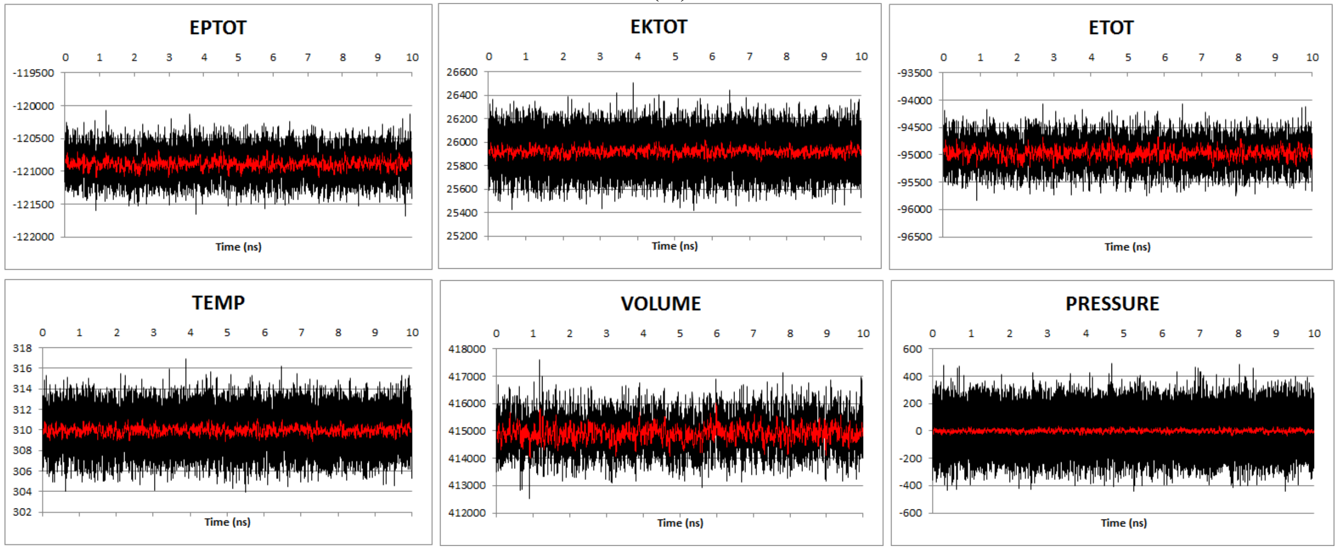

Pairwise Decomposition of Binding Energy

It is interesting to note that R394 gave a repulsion force to FevA which was indicated by its positive values of energy, contributed mostly by electrostatic energy. R394 is known to stabilize E2 along with E353. The structural investigation of four MD systems' average structure revealed that the proton from the hydroxyl group of E2 was quite far from the guanidino's hydrogens of R394 compared to the proton of the hydroxyl group of FevA and 4-OHT. The short distances between these two positive protons might have resulted in the repulsion. T347 also interacted well with FevA. Figure 9D demonstrated that the hydroxyl group of T347's side chain interacted with the oxygen carbonyl at position C22 of FevA. However, this interaction did not satisfy the 3.0 Å of distance and 120° of angle cutoffs to be classified as hydrogen bond. (see Table 1).

© 2014 by the authors; licensee MDPI, Basel, Switzerland. This article is an open access article distributed under the terms and conditions of the Creative Commons Attribution license (http://creativecommons.org/licenses/by/3.0/).
